# MUSCLE STRENGTH AND STIFFNESS OF ELBOW MUSCLES: CORRELATION WITH UPPER LIMB MOTOR FUNCTIONS IN PEOPLE WITH CHRONIC STROKE

**DOI:** 10.2340/jrm.v57.44075

**Published:** 2025-10-26

**Authors:** Shamay S.M. NG, Peiming CHEN, Shun Hei CHOI, Tung Ji LAM, Hei Yee LAU, Hing Ki LAU, Ho Yeung LAW, Desmond Y.W. LAM, Cynthia Y.Y. LAI

**Affiliations:** 1 Department of Rehabilitation Sciences, The Hong Kong Polytechnic University, Hung Hom, Hong Kong SAR; 2 Research Centre for Chinese Medicine Innovation, The Hong Kong Polytechnic University, Hung Hom, Hong Kong SAR; 3 Physiotherapy Clinic, Hong Kong, China

**Keywords:** stroke, upper limb, assessment

## Abstract

**Objective:**

To (*i*) quantify elbow flexor and extensor strength and stiffness in people with stroke; (*ii*) compare affected and unaffected sides; (*iii*) compare stroke survivors and healthy older adults; and (*iv*) examine correlations between muscle properties, motor control, and ADLs.

**Design:**

Cross-sectional study.

**Participants:**

65 stroke survivors and 31 healthy older adults.

**Methods:**

Elbow muscle strength and stiffness (biceps and triceps) were assessed bilaterally by Myoton PRO. Stroke participants also completed the Fugl-Meyer Assessment–Upper Extremity (FMA-UE), Action Research Arm Test (ARAT), Disabilities of the Arm, Shoulder and Hand (DASH), and Oxford Participation and Activity Questionnaire (OxPAQ).

**Results:**

Stroke participants had significantly weaker elbow muscles on the affected side (*p* < 0.001) than the unaffected side, but stiffness did not differ significantly. Compared with healthy adults, stroke participants showed reduced strength but similar stiffness. Weak to moderate correlations were found between muscle strength and FMA-UE, ARAT, and DASH (ρ = 0.336–0.613), but not with OxPAQ. Weak negative correlations were found between biceps stiffness and motor function (FMA, ARAT) (ρ = –0.343 to –0.397), and a weak negative correlation between triceps stiffness and OxPAQ emotional well-being (ρ = –0.313).

**Conclusion:**

Stroke survivors have reduced elbow strength but similar stiffness compared with the healthy elderly. Strength correlates moderately with upper limb function; stiffness shows inconsistent associations.

## LAY ABSTRACT

This study investigated the strength and stiffness of elbow muscles in people who had had a stroke, compared with healthy older adults. The muscle strength and stiffness were assessed in both arms, and how well people with stroke could move and use their arms in daily life was also tested. We found that the muscles on the affected side after stroke were much weaker than on the unaffected side. However, the stiffness of the muscles was similar between the two sides. Compared with healthy people, people with stroke had weaker muscles, but similar stiffness. In summary, stronger muscles were associated with better arm movement and function in daily tasks. Muscle stiffness, however, was not clearly related to how well people could use their arms. Stroke affects muscle strength more than stiffness, and improving strength may help with better arm use in daily life.

Stroke is a common neurological condition affecting 25% of the global population and often results in varying degrees of functional disability (1). In particular, up to 55% of people with chronic stroke have upper extremity motor impairments (2). Moreover, abnormal muscle stiffness and muscle weakness are the most prevalent motor impairments in people with stroke and profoundly hinder their functional performance in activities of daily living (ADLs) (3, 4).

Muscle weakness manifests as a reduction in the amount of force generated during muscle contraction (5) and an inability of muscles to generate the optimal force at the optimal range (6). It was previously found that approximately 70% of people with stroke in developing countries had upper limb muscle weakness that impeded their ability to perform ADLs (4). Post-stroke muscle weakness, or paresis, is primarily caused by brain damage, which leads to impaired motor control. Secondary sarcopenia, a condition characterized by the progressive loss of muscle mass and strength, further contributes to muscle weakness through 3 main mechanisms: the loss of motor units, a decrease in motor unit firing rate, and alterations in the neurophysiological properties of motor units (7).

Muscle stiffness manifests as an increase in the mechanical resistance of a muscle reacting to external perturbation. Approximately 40% of people with stroke were reported to experience increased muscle stiffness in the upper limbs (3), and such muscle stiffness is attributable to reflex-mediated and non-reflex-mediated mechanisms (8). The reflex-mediated mechanism after stroke is due to the imbalanced interaction between impaired descending facilitatory and inhibitory pathways of spinal stretch reflexes, resulting in hyperexcitability of the stretch reflex and increased muscle stiffness. This phenomenon, commonly referred to as spasticity, is characterized by a velocity-dependent increase in muscle tone, where resistance to passive movement becomes greater as the speed of the stretch increases. Spasticity arises from stroke-induced disruptions in central nervous system control, leading to impaired modulation of spinal reflexes and exaggerated stretch reflex responses (8). The non-reflex mediated mechanism is due to the structural changes in the contractile and the passive tissue of skeletal muscles. These changes include increased collagen deposition (9) and increased apoptotic activation, resulting in increased fat tissues (10); reduced fascicle length, resulting in reduced number of sarcomeres (11); and a shift in muscle fibre types in the muscles of the affected side (12).

Muscle stiffness and muscle weakness are interrelated. Gray et al. (6) proposed that muscles in a shortened position produce less force at lower velocity during contractions. Additionally, stiffened muscles undergo structural changes and thereby become immobilized in a shortened position. As a result, the amount and speed of force generation by stiffened muscles decreases, leading to further muscle weakness. Increased stiffness and weakness of upper limb muscles, for example, biceps brachii and triceps brachii, causes an imbalance in elbow movement patterns and improper alignment of the elbow joint, which hinders the performance of ADLs such as eating and dressing independently (13).

The biceps brachii and triceps brachii are the largest pair of agonists and antagonists in the upper limbs and thus their optimal coordination supports upper-limb motor functions involved in many ADLs. Elbow muscle weakness caused by the limited range of motion of the elbow and distal instability is found in people with stroke (14). Hence, they may be unable to perform repeated reaching movements, such as eating.

One of the important goals of stroke rehabilitation is to improve upper-limb motor function and thus enhance the independence of people with stroke in performing ADLs. To the best of our knowledge, there no previous study has investigated the correlation of upper limb muscle strength and stiffness simultaneously with the upper limb motor function and performance of ADLs in people with chronic stroke. Thus, the objectives of this study were to (*i*) compare the strength and stiffness of the elbow flexors and extensors in people with stroke with the strength and stiffness of these muscles in healthy older adults; and (*ii*) calculate the correlation between the muscle strength and muscle stiffness of the elbow muscles in people with stroke, the motor function of their upper limbs, and their participation in ADLs.

## METHODS

### Study design

This study used a cross-sectional design and was ethically approved by the Hong Kong Polytechnic University. Prior to the start of the study, its procedures and objectives were clearly and comprehensively explained to the participants and their informed consent was obtained. In addition, this study was conducted in accordance with the Declaration of Helsinki.

### Sample size calculation

No previous studies have investigated the correlation between the properties of the elbow flexors and extensors and their performance of ADLs in people with stroke. Thus, our sample size estimation was based on Chuang et al. (15), who found a significant correlation (ρ = 0.35) between pinch strength and muscle stiffness in people with stroke. We determined via calculation in G*Power 3.1.9.7 (Franz Faul, University of Kiel, Kiel, Germany) that a sample size of more than 59 would be required to obtain a statistically significant difference at a significance level of 0.05 and power of 80%. To ensure a robust finding, we increased the sample size to 65.

### Participants

Sixty-five people with stroke were recruited as participants and comprised the stroke group. The inclusion criteria for the stroke group were: (*i*) aged 50–80; (*ii*) diagnosed with stroke by computed tomography or magnetic resonance imaging at least 1 year ago; (*iii*) have minimal upper-limb function on the affected arm, although shoulder, elbow, and wrist muscles achieve grade 3 in manual muscle testing; (*iv*) had a score of at least 7 in the Abbreviated Mental Test (AMT); (*v*) able to sign the informed consent form; and (*vi*) have no other cardiovascular, neurological, or musculoskeletal diseases that may affect the performance or results of the assessments. The exclusion criteria for the stroke group were (*i*) have receptive dysphasia; (*ii*) have an orthopaedic or medical condition that would hinder proper assessment of the muscle property and functional performance.

In addition, 31 healthy older adults were recruited as participants and comprised the healthy group. These participants were aged 50–80, had no comorbidities that would affect their performance in the assessments, and met all the other inclusion and exclusion criteria mentioned above except for a stroke history.

### Assessment procedures

Assessments were conducted in a university-based rehabilitation laboratory. All participants were asked to give their written informed consent and complete a questionnaire to provide their demographic data. Subsequently, the affected and unaffected arm of people with stroke and the dominant and non-dominant arm of the healthy older adult were subjected to myotonometer and muscle strength assessment. In addition, the stroke group was assessed on the same day using the Fugl-Meyer Assessment – Upper Extremity (FMA-UE), the Action Research Arm Test (ARAT), the Disabilities of the Arm, Shoulder and Hand (DASH) questionnaire, and the Oxford Participation and Activity Questionnaire (OxPAQ).

### Measurement of muscle properties

*Measurement of maximal isometric muscle strength.* The strength of the elbow flexors and extensors was assessed using a hand-held dynamometer (HHD) (model 01165A, Lafayette Instrument Company, Lafayette, IN, USA), a battery-operated device that measures peak force in kilograms. Measurements were performed with the participants in a supine position. The positioning of the dynamometer, procedure, and verbal instructions used during the measurement of muscle strength in the upper limbs are presented in Appendix S1. The reliability of hand-held dynamometry for assessment of the upper-limb strength of people with stroke has not been determined, but it was previously found to have good to excellent test–retest reliability (intraclass correlation coefficient (ICC) = 0.75) for assessment of isometric lower-limb strength in healthy adults (16). The ankle muscle strength measured by HHD was also shown moderate to high correlation (*r* = 0.60–0.73) with the 6 Minute Walk Test performance in people with stroke.

**Fig. 1:**
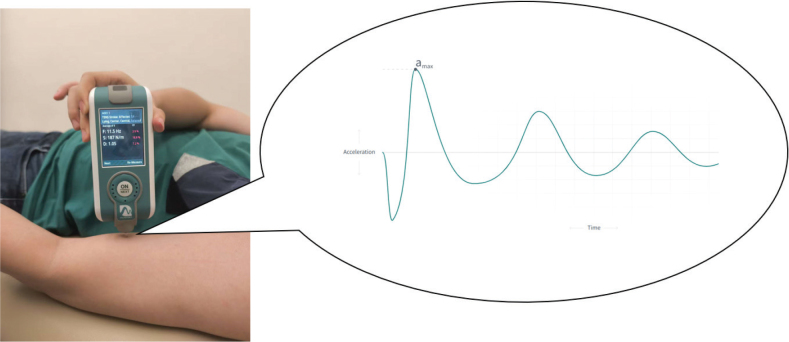
Mechanism to assess the muscle stiffness of the upper limb.

*Measurement of muscle stiffness.* Muscle stiffness was assessed using the MyotonPRO (MyotonAS, Tallinn, Estonia and MyotonLtd, London, UK), a hand-held myotonometer (17). Muscle stiffness (N/m) represents the resistance of biological soft tissue to a force of deformation. The formula that is used to calculate muscle stiffness is as follows: $S=\frac{\;\alpha_{max}\cdot m_{probe}}{l}$. The *α_max_* is the maximum acceleration of the probe, *m_probe_*, is the mass of the probe and these 2 factors are divided by Δ*l*, which is the maximum displacement of the tissue (Fig. 1) (17).

In the current study, we measured the muscle stiffness of the participants’ biceps brachii and triceps brachii in a relaxed sitting position. The Myoton device applies a brief mechanical impulse to the surface of the skin over the muscle. It then measures the tissu’s response to this impulse (18). The stiffness value is derived from the resistance of the tissue to the applied force. Five impulses were generated by a plastic stick inside the Myoton machine and record the reaction force from the tissue, and the resulting myotonometric measurements for each participant were averaged. The MyotonPRO achieved ICCs for stiffness of 0.90 and 0.88 in biceps brachii and triceps brachii respectively in people with stroke, which are considered to indicate high reliability (19). The upper limb muscle stiffness measured by MyotonPRO also showed a significant correlation (*r* = –0.35–0.57)) with the grip strength, pinch strength, and ARAT score in people with stroke (15)

*Fugl-Meyer Assessment of Upper Extremity (FMA-UE).* The FMA-UE is commonly used to assess upper-limb motor function (20). It employs a 3-point ordinal scale with 17 items to evaluate the movements, coordination of the shoulder, elbow, forearm, wrist, and hand, and the reflex activity of upper limb (21). The total score is 34. It was previously shown to have excellent intra-rater reliability (ICC = 0.99) and inter-rater reliability (ICC = 0.99) among stroke populations (22).

*Action Research Arm Test (ARAT).* The ARAT is designed to evaluate upper-limb movement related to activity in people with neurological problems. It consists of 19 items that assess grasping, gripping, pinching, and gross movements (23). Performance on each item is scored on a 4-point ordinal scale, with higher scores indicating better performance. The total score is 57. It was previously found that the ARAT has good test–retest reliability (ICC = 0.97) and internal consistency (Cronbach’s α = 0.96) in people with chronic stroke (24).

*Disabilities of the Arm, Shoulder and Hand (DASH).* The DASH questionnaire is a 30-item self-report questionnaire that measures assess musculoskeletal disorders of the upper limbs, (25). The total score is 100. Higher scores indicate a higher level of disability and symptom severity. The DASH questionnaire was shown to have good internal consistency (Cronbach’s α = 0.92) and fair test–retest reliability (ICC = 0.56) in the most recent psychometric study in a stroke population (26).

*The Oxford Participation and Activities Questionnaire (OxPAQ).* The OxPAQ is a 23-item, International Classification of Functioning, Disability and Health (ICF)-model-based instrument that is used to assess participation and activity levels (27). Scores are allocated in 3 domains (routine activities, social engagement, and emotional well-being). The total score was 100. A higher score indicates a higher level of participation restriction in the activity and participation component of the ICF model. The Chinese version of the Ox-PAQ-C was previously shown to have high validity and reliability (ICC = 0.91–0.94) in a stroke population (28).

### Statistical analysis

Data were analysed using Statistical Product and Service Solutions software version 29.0.0.0 (IBM Corp, Armonk, NY, USA). A *p*-value of less than 0.05 was regarded as a statistically significant difference in the demographic information and performance.

Descriptive statistics was used to summarize the demographic characteristics, the muscle properties, and the results of other outcome measures. The Kolmogorov–Smirnov test was used to assess the normality of data.

An independent samples *t-*test was conducted to analyse the differences in muscle properties between the stroke group and the healthy group. A paired samples *t*-test was used to analyse the differences in muscle properties between the affected arm and unaffected arm of the stroke group. Bonferroni correction was used to adjust the significant level when performing the multiple comparison.

Spearman’s ρ test was conducted to assess the correlations between muscle properties and different outcome measures, with pairwise deletion used for missing data. Thus, correlation coefficients lower than or equal to 0.30, 0.30–0.50, 0.50–0.70, 0.70–0.90, and greater than 0.90 were regarded as weak, low, moderate, strong, and very strong relationships, respectively (29).

## RESULTS

Table I presents the demographic information and the performance under different outcomes. The stroke group comprised 65 participants with chronic stroke (35 men and 30 women), and the healthy group comprised 31 healthy participants (17 men and 14 women). The mean age (standard deviation) of the stroke group was 66.91 (6.40) years, and for the healthy group this was 64.84 (7.85) years. In the stroke group, the mean time since stroke was 9.20 years (5.01), and 40 had ischaemic stroke, 24 had haemorrhagic stroke, and 1 had both types of strokes.

**Table I:** Demographics and performance of participants

Characteristics	Stroke (*n* = 65)Mean (SD)	Healthy (*n* = 31)Mean (SD)	*p*-value
Age, years	66.91 (6.40)	64.84 (7.85)	0.207
Gender, male/female	35/30	17/14	1.000
Height, cm	162.30 (7.93)	164.93 (8.79)	0.149
Weight, kg	63.25 (10.37)	64.63 (11.70)	0.567
BMI, kg/m^2^	24.03 (3.38)	23.77 (4.09)	0.742
Time since stroke, years	9.20 (5.01)	N/A	N/A
Affected side, left/right, *n*	31/34	N/A	N/A
Type of stroke, ischaemia/haemorrhage/both, *n*	40/24/1	N/A	N/A
FMA-UE	45.31 (17.16)	N/A	N/A
ARAT	32.92 (24.15)	N/A	N/A
DASH	27.54 (9.57)	N/A	N/A
OxPAQ RA	19.22 (19.21)	N/A	N/A
OxPAQ SE	11.53 (17.30)	N/A	N/A
OxPAQ EW	18.36 (20.26)	N/A	N/A

SD: standard deviation; BMI: body mass index; N/A: not applicable; ARAT: Action Research Arm Test; DASH: Disabilities of Arm; Shoulder; and Hand; FMA-UE: Fugel-Meyer Assessment of Upper Extremity; OxPAQ: Oxford Participation and Activities Questionnaire; RA: routine activities; SE: social engagement; EW: emotional well-being.

Table II compares the muscle properties between the affected and unaffected arm in the stroke group. There was a significant difference in elbow muscle strength between the affected and unaffected arms (*p* < 0.001). However, there was no significant difference in elbow muscle stiffness between the affected and unaffected arms.

**Table II:** Comparison of muscle properties between affected arm and unaffected arm of people with stroke

Muscle properties	Affected armMean (SD)	Unaffected armMean (SD)	*p*-value
Muscle stiffness			
BB Stiffness, N/m	184.86 (58.45)	171.85 (36.38)	0.066
TB Stiffness, N/m	180.06 (43.42)	171.15 (41.88)	0.059
Muscle strength			
Elbow flexors, kg	8.62 (4.00)	12.71 (3.58)	< 0.001*
Elbow extensor, kg	7.20 (3.32)	10.59 (3.13)	< 0.001*

SD: standard deviation; BB: biceps brachii; TB: triceps brachii.

* *p* < 0.05/4 = 0.0125 after Bonferroni correction.

Table III compares the muscle properties between the stroke group and the healthy group. As can be seen, there was no significant difference between the elbow muscle stiffness of the affected arm of the stroke group and that of the non-dominant arm of the healthy group. However, there was a significant between-group -difference in elbow muscle strength (*p* < 0.001). In addition, the biceps brachii stiffness of the unaffected arm of the stroke group was significantly lower than that of the dominant arm of the healthy group (*p* = 0.006). There was no significant difference between the triceps brachii stiffness in the unaffected arm of the stroke group and that of the dominant arm of the healthy group. Finally, there was significant between-group difference in elbow flexion strength (*p* < 0.001).

**Table III:** Comparison of muscle properties between people with stroke and healthy older adults

Muscle properties	StrokeMean (SD)	HealthyMean (SD)	*p*-value
	Affected arm	Non-dominant arm	
Muscle stiffness			
BB Stiffness, N/m	184.86 (58.45)	203.07 (42.55)	0.135
TB Stiffness, N/m	180.06 (43.42)	173.24 (40.00)	0.473
Muscle strength			
Elbow flexor, kg	8.62 (4.00)	13.96 (4.07)	< 0.001*
Elbow extensor, kg	7.20 (3.32)	10.52 (3.07)	< 0.001*
	Unaffected arm	Dominant arm	
Muscle stiffness			
BB stiffness, N/m	171.85 (36.38)	197.83 (50.36)	0.006*
TB stiffness, N/m	171.15 (41.88)	179.97 (40.42)	0.343
Muscle strength			
Elbow flexor, kg	12.71 (3.58)	14.84 (4.78)	0.017
Elbow extensor, kg	10.59 (3.13)	10.97 (3.70)	0.574

SD: standard deviation; BB: biceps brachii; TB: triceps brachii.

* *p* < 0.05/8 = 0.006 after Bonferroni correction.

Table IV indicates the correlations between muscle properties and stroke-specific outcome measures. A fair to moderate correlation (ρ = –0.388 to 0.613) was found between the elbow muscle strength and all the stroke-specific outcomes, except for OxPAQ. Only the DASH questionnaire score had a negative correlation (ρ = –0.336 to –0.388) with muscle strength. The FMA-UE score had moderate to good correlation (ρ = 0.516 to 0.613) with biceps and triceps brachii muscle strength. Individual analysis of the 3 domains of the OxPAQ revealed that elbow flexion strength was significantly and negatively correlated with the routine activities domain, although the correlation was weak (ρ = –0.304). The remaining domains of the OxPAQ were not significantly correlated with the elbow muscle strength.

**Table IV:** Correlations between muscle properties and stroke-specific outcome measures

Muscle properties	FMA-UE	ARAT	DASH	OxPAQ RA	OxPAQ SE	OxPAQ EW
Muscle stiffness						
BB stiffness, N/m	–0.343 (*p* = 0.005*)	–0.397 (*p* = 0.001*)	0.198 (*p* = 0.114)	0.013 (*p* = 0.920)	–0.057 (*p* = 0.653)	–0.205 (*p* = 0.104)
TB sStiffness, N/m	–0.118 (*p* = 0.351)	–0.108 (*p* = 0.392)	–0.109 (*p* = 0.388)	–0.232 (*p* = 0.066)	–0.134 (*p* = 0.290)	–0.313 (*p* = 0.012*)
Muscle strength						
Elbow flexor, kg	0.613 (*p* < 0.001*)	0.513 (*p* < 0.001*)	–0.388 (*p* = 0.002*)	–0.304 (*p* = 0.015*)	–0.154 (*p* = 0.227)	–0.034 (*p* = 0.790)
Elbow extensor, kg	0.516 (*p* < 0.001*)	0.410 (*p* < 0.001*)	–0.336 (*p* = 0.007*)	–0.165 (*p* = 0.195)	–0.006 (*p* = 0.963)	0.088 (*p* = 0.493)

SD: standard deviation; BB: biceps brachii; TB: triceps brachii; FMA-UE: Fugel-Meyer Assessment of Upper Extremity; ARAT: Action Research Arm Test; DASH: Disabilities of Arm, Shoulder, and Hand; OxPAQ: Oxford Participation and Activities Questionnaire; RA: routine activities; SE: social engagement; EW: emotional well-being.

* p<0.05

Significant, negative, and low correlations were found between biceps brachii stiffness and the outcomes assessed by the FMA and ARAT (ρ = –0.343 to –0.397). A significant and low negative correlation was found between triceps brachii stiffness and the emotional well-being domain of the OxPAQ (ρ = –0.313).

## DISCUSSION

The key advantage of this study is the simultaneous assessment of both muscle strength and muscle stiffness in relation to upper limb motor function in people with stroke. This dual approach provides a more comprehensive understanding of how these distinct muscle properties collectively influence functional performance. The biceps brachii and triceps brachii in the affected arm of the stroke group were significantly weaker than the non-dominant arm of the healthy groups. The biceps brachii, but not the triceps brachii, were significantly weaker in the unaffected arm of the stroke group than in the dominant arm of the healthy group. The stiffness of the biceps brachii and triceps brachii in the affected arm of the stroke group was not significantly different from that of the non-dominant arm of the healthy group. The muscle stiffness of the biceps brachii, but not that of the triceps brachii, in the unaffected arm of the stroke group was significantly different than the biceps brachii in the dominant arm of the healthy group. The strength of the biceps brachii and triceps brachii in the affected arm of the stroke group was significantly correlated with FMA-UE, ARAT, and DASH questionnaire scores. The stiffness of the biceps brachii, but not that of the triceps brachii, in the affected arm of the stroke group was significantly correlated with FMA-UE and ARAT scores.

### Decreased muscle strength on the affected arm

Our findings suggest that the muscles in the affected arm of the stroke group were weaker than those in the non-dominant arm of the healthy group, consistent with previous research (30). Post-stroke muscle weakness is a multifactorial phenomenon caused by primary neurological changes, learned non-use, and ageing. Neurological changes, such as reduced descending drive from the motor cortex and over-reliance on alternative descending pathways (e.g., reticulospinal tracts), contribute to muscle denervation, abnormal co-contraction of agonist and antagonist muscles, and impaired ability to relax or generate maximal strength (5, 15, 31). Learned non-use develops as patients avoid using their affected arm due to pain, paresis, or limited range of motion in the early recovery phase, which leads to muscle atrophy and reduced voluntary activation over time (7, 32). Additionally, ageing may exacerbate muscle weakness, as evidenced by the 2-year higher mean age of the stroke group compared with the healthy group in this study. Age-related muscle changes, including increased intermuscular adipose tissue, reduced muscle mass, and smaller type II muscle fibres, are well-documented contributors to declining muscle strength, particularly in individuals over 50 years old (33, 34).

Our findings suggest that the muscles in the affected arm of the stroke group were weaker than those in the non-dominant arm of the healthy group, which is consistent with the findings of a previous study (30). Post-stroke muscle weakness is caused by multiple mechanisms, including primary neurological changes, learnt non-use, and ageing. These mechanisms are described below.

### Decreased muscle strength of the unaffected arm

Consistent with the finding of previous studies (35), the results of the current study demonstrated that only the biceps brachii in the unaffected arm of the stroke group were weaker than those in the dominant arm of the healthy group, and almost reach a significant level. The muscle weakness on the unaffected arm was associated with the corticospinal tract projection. A previous animal study had found that approximately 10% of the corticospinal tract descends to the ipsilateral side (36), suggesting that a stroke-generated lesion in the cerebral hemisphere also influenced the ipsilesional unaffected arm. Nevertheless, given that less of the corticospinal tract was present on the unaffected arm than on the affected arm, the muscles on the unaffected arm were less weakened than that on the affected arm. The aforementioned relationship was also apparent in the current study and is partly attributable to the higher usage of the unaffected arm than of the affected arm. For example, it was previously demonstrated that, compared with the affected arm, the unaffected arm was used 3–6 times more in a normal living environment (37). Consequently, the primary deficit was better recovered in the unaffected arm. This may account for the non-significant decrease in elbow extensor strength observed in the stroke group when compared with the healthy group in this study.

### Increased muscle stiffness of the affected arm

Surprisingly, findings of the current study revealed a nonsignificant difference in muscle stiffness between the arm of people with stroke and the arm of healthy older adults. This finding differs from previous studies that showed upper limb muscle stiffness is significantly increased in people with stroke (38). The increase in muscle stiffness is associated with multiple factors, such as changes in the type of muscle fibres, decreased fascicle lengths, and increased content of connective tissues in the extracellular matrix.

People with stroke are observed to have an increased proportion of type II fibres in muscle (12). This shift in fibre type proportion is due to the higher sensitivity to the decreased neural activity in type I fibres, inducing a higher proportion of atrophy after denervation and immobilization (39). Hence, the relative proportion of type II fibres will increase in the muscles of people with stroke. As type II fibres have a higher passive tension than type I fibres (40), the increase in proportion of type II fibres will lead to increased muscle stiffness in people with stroke.

Decreased fascicle length of muscle fibres has been observed in the affected limbs of people with stroke, with this observation also correlating with increased muscle stiffness (11). The decrease in fascicle length has been suggested to be a result of the shortened positioning of muscle, as shown by previous animal work (41). After stroke, the impairments and disuse of the affected limbs may lead to reduced excursion of muscles in these limbs, resulting in an adaptive decrease in fascicle length.

Interestingly, the biceps brachii stiffness of the unaffected arm in the stroke group was found to be significantly lower than that of the dominant arm in the healthy group. It is important to note that the muscle stiffness measured by the MYOTON device reflects the mechanical properties of the muscle (17), such as viscoelasticity, rather than spasticity (8), which is a velocity-dependent increase in muscle tone due to hyperactive stretch reflexes. While brain lesions in people with stroke may impair muscle properties in the unaffected limb, there is no consensus on whether this leads to increased or decreased muscle stiffness. Additionally, our inclusion criteria did not specify the muscle stiffness level of the stroke group, which may have contributed to the observed variability. In our study, 66.2% of stroke participants (43 out of 65) exhibited lower muscle stiffness in the unaffected arm compared with the mean stiffness of the healthy group, suggesting significant variability in muscle stiffness among stroke patients. This variability may be influenced by individual differences in muscle properties, the extent of motor impairment, and the ability to relax during measurements. These findings highlight the need for further studies with larger sample sizes and standardized methodologies to better understand the muscle stiffness characteristics of the unaffected limb in stroke populations.

The non-significant findings on the biceps brachii and triceps brachii stiffness in the current study may be due to the method used to measure muscle stiffness. Most previous studies have measured stiffness via ultrasonography (38), whereas we measured with myotonometry. While ultrasound image can differentiate the muscle from surrounding structures, the myotonometric measurement relies on the probe over the skin, hence the subcutaneous fat thickness will also contribute to the stiffness measured by myotonometry (42). Second, unlike previous studies, the stroke group in the current study was recruited from a self-help group. The recruited individuals may have more active lifestyles with higher independence in daily activities, hence their likelihood to have impaired muscle stiffness may be lower. As a result, the findings of the current study are different from the previous studies.

### Correlation between muscle properties and functional ability

The results of the current study demonstrated that in people with stroke, the biceps brachii and triceps brachii strength is significantly correlated with upper limb motor function. This finding aligns with those of previous studies, which have found that upper limb strength is significantly correlated with functional outcome (4). The correlation is due to the requirement of the biceps and triceps brachii generating sufficient force to perform bimanual actions and to provide proximal stability during fine hand movements. Hence, the decrease in biceps brachii and triceps brachii strength in people with stroke would directly affect their performance in upper limb functional tasks.

However, there was generally no significant correlation between muscle strength and the domains of OxPAQ in the current study. The lack of correlation is attributable to the multifactorial nature of activity and participation, diminishing the impact of muscle strength in OxPAQ outcome. Regarding the intrinsic factors, individual coping strategies are crucial to behavioural adaptations. The changes of behaviour assist people with stroke to overcome functional challenges, thus promoting their social participation (43). Regarding the extrinsic factors, financial support, assistive technologies, and accessible amenities eliminate physical barriers for people with stroke to social participation. Hence, muscle strength of people with stroke is not the sole factor of activity and participation.

Given that muscle weakness and muscle stiffness are interrelated impairments after stroke (6), we expected that the correlation of upper limb motor function with muscle stiffness would be similar to that with muscle strength. Surprisingly, the findings of the current study demonstrated inconsistent correlations between upper limb motor function with biceps brachii and triceps brachii stiffness. This is contrary to the previous studies, which showed significant correlations between upper limb muscle stiffness and functional outcomes (15). One possible explanation is that the muscle stiffness was measured at rest, while previous studies measured muscle stiffness during contraction. The muscle stiffness significantly increases as the contraction level increases, showing that the active component also contributes significantly to the total stiffness (38). Future research could investigate the correlation between functional outcomes and the muscle stiffness of contracted muscles, which may better reflect muscle stiffness during functional tasks.

### Limitations

First, our findings may be limited by the small sample size. Thus, while the sample size was sufficient to detect significant correlations, a larger cohort would help reduce individual variability and provide more robust findings, particularly for the unexpected muscle stiffness results, which could be influenced by individual variability. A larger sample size would help to reduce this variability, provide more robust findings, and enable subgroup analyses to explore additional factors, such as stroke type, severity, and rehabilitation history, which may influence muscle stiffness. Second, our findings might have been influenced by the sex differences as both stroke and healthy group contained more men than women. Differences in muscle strength and motor task performance between opposite sexes may affect muscle strength, muscle stiffness, and stroke-specific outcomes (44). Third, we measured isometric muscle strength and muscle stiffness in a resting position, which cannot fully reflect the muscle force generated and muscle stiffness during dynamic movements in ADLs. Thus, future studies should measure isokinetic muscle strength and muscle stiffness at various joint positions during ADLs. Fourth, the technical limitations of MyotonPRO are the inability to measure the stiffness of small muscles or those covered by greater than 2 cm of subcutaneous fat (42). Fifth, although the hand-held dynamometer has shown excellent reliability when assessing the upper limb muscle strength in healthy adults, it was not validated to assess the upper limb muscle strength in people with stroke. As the stroke population showed muscle weakness, spasticity, abnormal movement patterns, and compensatory strategies when compared with healthy adults, the interpretation of our strength measurements must be approached with caution. Further research should establish the reliability and validity of hand-held dynamometry specifically for individuals with stroke-related upper limb impairments. Lastly, we examined only the muscle strength and muscle stiffness in the elbow. However, the study did not examine shoulder gross motor function and hand fine motor function (e.g., grasp, grip, and pinch), which are essential functions for activities of daily living. Future studies should also examine shoulder or hand muscles to deduce possible correlation between upper-limb muscle properties and functional outcomes in people with stroke.

### Conclusion

This study showed that the strength of elbow flexors and extensors in the stroke group was weaker than those of the healthy group. However, there was insignificant difference between the stiffness of the elbow flexors and extensors in the stroke group compared with that of the healthy group. In the stroke group, there was a weak-to-moderate correlation between the strength of elbow flexors and extensors and FMA-UE, ARAT, and DASH, but no correlation between the muscle strength and OxPAQ domains. In addition, in the stroke group, there was an inconsistent correlation between the stiffness of elbow muscles and upper limb motor function.

## Supplementary Material


