# Supplementary material for: MUSCLE STRENGTH AND STIFFNESS OF ELBOW MUSCLES: CORRELATION WITH UPPER LIMB MOTOR FUNCTIONS IN PEOPLE WITH CHRONIC STROKE
Source: J Rehabil Med. 2025 Oct 26;57:44075. doi: 10.2340/jrm.v57.44075 (PMC12576853; doi:10.2340/jrm.v57.44075)
Supplement: Supplementary file 1 [file JRM-57-44075-s1.pdf]

Supplementary material has been published as submitted. It has not been copyedited, or typeset by Journal of Rehabilitation Medicine

Muscle stiffness measurement

Instruction to patients	Biceps brachii:<br>'Please lie down, with the elbow flexed to 90 degrees and shoulder abducted to 45 degrees'<br><br>Triceps brachii:<br>'Please sit down, with shoulder and elbow in neutral orientation'
Placement of MyotonPRO probe	Biceps brachii: Perpendicularly on the lower 33% of the area between the coracoid process of the scapula and the radial side of the cubital fossa<br><br>Triceps brachii: Perpendicularly on the midpoint between the acromion and olecranon.
Values to be recorded	A short impulse is generated, followed by a quick release of the probe. The device will generate 5 impulses and the average scores of myotonometric measurements will be taken. Muscle stiffness (N/m) will be recorded for once for biceps brachii, triceps brachii
